# Assessing Genetic Diversity and Estimating the Inbreeding Effect on Economic Traits of Inner Mongolia White Cashmere Goats Through Pedigree Analysis

**DOI:** 10.3389/fvets.2021.665872

**Published:** 2021-06-22

**Authors:** Zhiying Wang, Bohan Zhou, Tao Zhang, Xiaochun Yan, Yongsheng Yu, Jinquan Li, Bujun Mei, Zhixin Wang, Yanjun Zhang, Ruijun Wang, Qi Lv, Zhihong Liu, Yanhong Zhao, Chen Du, Rui Su

**Affiliations:** ^1^College of Animal Science, Inner Mongolia Agricultural University, Hohhot, China; ^2^Inner Mongolia Bigvet Co., Ltd., Hohhot, China; ^3^Key Laboratory of Mutton Sheep Genetics and Breeding, Ministry of Agriculture, Hohhot, China; ^4^Key Laboratory of Animal Genetics, Breeding and Reproduction in Inner Mongolia Autonomous Region, Hohhot, China; ^5^Engineering Research Center for Goat Genetics and Breeding, Inner Mongolia Agriculture University, Hohhot, China; ^6^Department of Agriculture, Hetao College, Hetao University, Bayannaoer, China; ^7^Reproductive Medicine Center, Affiliated Hospital of Inner Mongolia Medical University, Hohhot, China

**Keywords:** inner Mongolia white cashmere goats, genetic diversity, regression analysis, population structure, inbreeding

## Abstract

**Objective:** The purpose of this study was to discover the population structure and genetic diversity of Inner Mongolia White Cashmere goats (IMCGs) and demonstrate the effect of inbreeding on the live body weight (LBW), cashmere yield (CY), fiber length (FL), and fiber diameter (FD) of IMCGs.

**Materials and Methods:** All data were collected from pedigree information and production performance records of IMCGs from 1983 to 2019. The population structure and genetic diversity were analyzed by Endog 4.8 software. Inbreeding coefficients were obtained by the pedigree package in R. Then, a linear regression model was used to analyze how inbreeding influences economic traits in IMCGs. Four levels of inbreeding coefficients (*F*_*i*_) were classified in this study, including *F*_*i*_ = 0, 0< *F*_*i*_ ≤ 6.25, 6.25< *F*_*i*_ ≤ 12.5 and *F*_*i*_≥12.5. Variance analysis was performed to determine whether inbreeding levels had a significant effect on economic traits in IMCGs.

**Results:** The proportions of rams and dams in IMCGs for breeding were relatively small, with values of 0.8 and 20.5%, respectively. The proportion of inbred animals in the entire population was high, with values up to 68.6%; however, the average inbreeding coefficient and relatedness coefficient were 4.50 and 8.48%, respectively. To date, the population has experienced 12 generations. The average generation interval obtained in the present study was 4.11 ± 0.01 years. The ram-to-son pathway was lowest (3.97 years), and the ewe-to-daughter pathway was highest (4.24 years). It was discovered that the LBW, CY, and FL increased by 3.88 kg, 208.7 g, and 1.151 cm, respectively, with every 1% increase in the inbreeding coefficient, and the FD decreased by 0.819 μm with every 1% increase in the inbreeding coefficient. Additionally, multiple comparison analysis indicated that when the inbreeding coefficient was higher than 6.25%, the LBW showed an obvious decreasing trend. The threshold value of inbreeding depression in the CY is 12.5%. However, inbreeding depression has not been observed in the FL and FD.

**Conclusion:** Pedigree completeness needs to be further strengthened. The degree of inbreeding in this flock should be properly controlled when designing breeding programs.

## Introduction

As a typical small ruminant, cashmere goats are mainly distributed in dry and harsh climatic conditions in the tropics (1). Goat farming is practiced worldwide, with goat products having a favorable image (2, 3). The number of goats has increased globally, even in countries with high and intermediate incomes, despite major changes in agriculture due to industrial mergers, globalization, and technological advances in developed countries (4, 5). Most cashmere goats are dual-purpose breeds that produce cashmere, meat, and milk (6). China is the largest cashmere goat-producing country in the world. According to statistics, the total cashmere produced in China was ~15,437.76 tons in 2018, accounting for more than 2/3 of the world's total output. The export volume of cashmere reached 3,212 tons (http://www.fao.org/home/en/). The genetic resources of cashmere goats are very rich throughout the world and are mainly distributed in Asia, including Mongolia, Iran, Afghanistan, Kazakhstan, Kyrgyzstan, and Tajikistan. Inner Mongolia White Cashmere goats (IMCGs) and Liaoning Cashmere goats (LNCGs) are key breeds in China (7). The male parents of most goat breeds in China come from both varieties (8). IMCGs and LNCGs are not allowed to be exported and are listed in the directory of genetic resource protection. The number of goat breeds in China is ~69, including 18 cashmere goat breeds. There are 15 local varieties and 3 cultivated varieties (9).

Genetic diversity and population structure in animals are usually analyzed by using microsatellite and mitochondrial DNA variations (10, 11). In recent years, pedigree analysis has been an appropriate tool for evaluating genetic diversity and population structure in populations (12–16). Baena et al. (12) analyzed the genetic structure of the Mangalarga Marchador horse population in Brazil based on pedigree analysis and identified factors that may affect its genetic variability. Illa et al. (14) assessed the genetic diversity and population structure and appraised the efficiency of ongoing selective breeding programs in a closed nuclear herd of Nellore sheep through pedigree analysis. Vigeland (16) calculated relatedness coefficients by using pedigree information with inbred founders. Vatankhah et al. (15) described the population genetic structure and evaluated the state of conservation of genetic variability of Lori-Bakhtiari sheep in Iran. Goleman et al. (13) analyzed the degree of relatedness between individuals in the Polish hunting dog population and assessed the genetic variability of the population based on pedigree analysis. The analysis of genetic diversity and population structure will contribute to the conservation of animal genetic resources (14). IMCGs are an indigenous breed that is famous around the world due to its superior cashmere. In a previous study, the genetic diversity and population structure of IMCGs were analyzed by mitochondrial DNA and microsatellite polymorphism (17). This is the first study to analyze the population structure and genetic diversity of IMCGs based on pedigree information.

Many studies have reported that inbreeding in one flock may result in a decrease in production performance (14, 18). Yousefi et al. (19) quantified the effect of inbreeding on the average daily gain and the Kleiber ratio in native Mazandaran chickens. It was demonstrated that inbreeding had a significant effect on the average daily gain from hatching to 8 and 12 weeks of age (19). Paiva R demonstrated that MY305 was significantly affected by inbreeding (20). Todd et al. (18) analyzed the effects of inbreeding on covering success, gestation length, and foal sex ratio in Australian thoroughbred horses. Kiya et al. (21) reported that the inbreeding effect was significant for the longissimus muscle area and backfat thickness. In this study, the effect of the inbreeding coefficient on important economic traits in IMCGs was analyzed, which is helpful for properly designing mating schemes.

## Materials and Methods

### Data Sources

In this study, genealogical information of IMCGs for a period of 37 years from 1983 to 2019 was collected for genetic diversity and population structure analysis. A total of 53,381 individuals were recorded, including 25,032 male lambs and 28,349 female lambs. The pedigree was edited to check the inconsistencies in dam and sire registration, birth date and sex registration. All of the individuals were sorted by date of birth for the next analysis.

The data were provided by Inner Mongolia Yiwei White Cashmere Goat Co., Ltd, China. The goats are reared at Etuoke Banner, Ordos City, Inner Mongolia Autonomous Region, China with arid and semiarid areas. IMCGs graze year round with supplementary feeding in the winter. Artificial insemination was used in this flock. The mating ratio of male to female sheep was ~1:200–300. Mating usually occurred in early October of each year and lasted nearly 50 days. Lambs were born during the month of March. The feeding management and trait measurement methods have been described in detail in our previous studies (22). All birthing events were recorded, including the identification number, date of birth, birth status, sex, and birth weight. Pedigree information was comprehensive and clear with kids, sires, and dams. The traits evaluated in this study included the live body weight (LBW), cashmere yield (CY), fiber length (FL), and fiber diameter (FD). The basic statistics for each trait are shown in Table 1.

**Table 1:** The basic statistics of economic traits of IMCGs.

**Traits**	***N***	**MEAN**	**SD**	**C.V(%)**
Live body weight	63,217	34.67	10.03	28.92
Cashmere yield	65,831	661.7	221.9	33.54
Fiber length	66,148	5.83	1.13	19.44
Fiber diameter	13,260	14.57	0.99	6.78

### Statistical Analysis

#### Analysis of Genetic Diversity and Population Structure

Endog 4.8 software was utilized to perform pedigree analysis and obtain corresponding parameters to illustrate the historical diversity and population structure (23). The depth and wholeness of the pedigree was determined by estimating the equivalent number of generations, and it was estimated by tracing back each ancestor in the pedigree history through numerous generations.

The founders are defined as individuals with one or both unknown parents. A 4-year time path was used to define the reference population because this time duration represents an approximate generation interval in goats (14). In the reference population, the effective number of founders and ancestors is useful to assess genetic diversity. The effective number of founders is characterized as the number of equally contributing founders that would be expected to produce the same genetic diversity as in the population under study (24). The formula is as follows:

$$f_{e}=\frac{1}{\sum_{k=1}^{f}q_{k}^{2}}$$

where *q*_*k*_ is the probability of gene origin for ancestor k. The effective number of ancestors (*f*_*a*_) reflects the minimum number of animals required to estimate the genetic diversity of the population under study, and it is a useful measure to determine the bottlenecks in the population that are the primary reason for genetic erosion in captive and domestic populations. It is estimated as:

$$f_{a}=\frac{1}{\sum_{j=1}^{a}q_{j}^{2}}$$

where *q*_*j*_ is the marginal contribution of ancestor j, which demonstrates the genetic contribution of an ancestor that is not explained by an earlier ancestor. In general, the effective number of ancestors should be smaller than the effective number of founders due to bottlenecks that reduce genetic variability.

The inbreeding coefficient (*F*) and the average relatedness (AR) coefficient were estimated by Meuwissen and Luo (24) and MaléCot (25), respectively. The AR coefficient of any animal is explained as the probability that an allele selected at random from the total population in the pedigree belongs to a particular animal; hence, it is equated as an account of the animal in the entire pedigree regardless of the pedigree information. *F* is defined as the probability that an individual has two identical alleles by descent. The change in breeding (Δ*F*) is estimated for each generation using the formulae suggested by Lacy (26).

$$\begin{array}{l} \text{Δ}F_{i}=1-\sqrt[{t-1}]{1-F_{i}} \end{array}$$

where *F*_*i*_ is the individual inbreeding coefficient and *t* is the equivalent complete generation for this individual. The estimate of effective population size ($N_{e}$) was computed from $\text{Δ}F_{i}$ by averaging Δ*F*_*i*_ of *n* individuals included in a given reference subpopulation (27) as $N_{e}=1/2\text{Δ}\bar{F}$.

The genetic conservation index (GCI) for each of the individuals of the analyzed population was provided by Alderson (28). The index is computed from the genetic contributions of all of the identified founders as $GCI=\frac{1}{\sum p_{i}^{2}}$, where *p*_*i*_ is the proportion of genes of founder i in the pedigree of an animal. The index is based on the assumption that the objective of a conservation program is to retain the full range of alleles possessed by the base population. In this respect, the ideal individual would receive equal contributions from all of the founder ancestors in the population, and consequently, the higher the GCI value is, the higher the values of an animal for conservation (28). The following parameters were estimated for each individual: (1) the number of fully outlined generations, detailed as the number of generations delineating the offspring of the furthest generation where the ancestors of second-generation individuals are known, and ancestors with unknown parents are considered founders (generation 0); (2) the maximum number of generations observed, determined as the number of generations separating the individual from its ultimate ancestor; and (3) equivalent complete generations are detailed as the sum over all known ancestors of the terms calculated as the aggregate of (1/2)^*n*^, where n is the number of generations separating the individual from each known ancestor. The average generation interval (GI) was pointed out as the average age of the birth of selected offspring. The estimate of GI for all of the pathways was estimated for the reference population, as this subpopulation is the most recent one that could accrue at least one generation on the farm.

Wright's (29) *F*-statistics are obtained as $F_{IS}=\frac{\tilde{F}-\bar{f}}{1-\bar{f}}$, $F_{ST}=\frac{\bar{f}-\tilde{f}}{1-\tilde{f}}=\frac{D}{1-\tilde{f}}$ and $F_{IT}=\frac{\tilde{F}-\tilde{f}}{1-\tilde{f}}$, where $\tilde{f}$ and $\tilde{F}$ are the mean coancestry and inbreeding coefficient for the entire metapopulation, respectively, and $\bar{f}$ is the average coancestry for the subpopulation. D is the kinship distance for molecular coancestry (30, 31).

The parameter of founder genome equivalents (*f*_*g*_) can be defined as the number of founders that would be expected to produce the same genetic diversity as in the population under study if the founders were equally represented and no loss of alleles occurred. Parameter *f*_*g*_ was obtained by the inverse of twice the average coancestry of the individuals included in a predefined reference population (30).

#### Effect of Inbreeding on Important Economic Traits of IMCGs

The basic statistics of each trait in this study are shown in **Table 3**. Except for the fiber diameter, the other three traits were collected from 1990 to 2019. The individual inbreeding coefficient was obtained by pedigree packages in R (32). A multiple linear regression model was used to analyze how the inbreeding coefficient influenced the four traits. In this study, the impact factors of each trait included the production year (1990–2019), flocks (1–12), age of individuals (1–7), age of dams (2–7) birth status (1, 2), and gender (1, 2). The inbreeding coefficient (*F*_*i*_) is continuously viable; however, other factors are discrete variables. Therefore, these discrete variables need to be centered first. Then, regression analysis was carried out by using the lm function of R language (33). The regression model is as follows:

$$\begin{array}{l} y_{i}=\gamma_{00}+\gamma_{01}Year_{i}+\gamma_{02}Flock_{i}+\gamma_{03}Age_{i}+\gamma_{04}Dage_{i}\\ \;+\gamma_{05}Sex_{i}+\gamma_{06}Bs_{i}+\gamma_{07}F_{i}+e_{i} \end{array}$$

where *y*_*i*_ is the vector of the observed value of the *i*^*th*^individual. *Year*_*i*_, *Flock*_*i*_, *Age*_*i*_, *Dage*_*i*_, *Sex*_*i*_, *Bs*_*i*_ , and *F*_*i*_ are independent variables, while *e*_*i*_ is the residual. γ_00_ is an intercept, which was also used as the overmean. γ_01_, γ_02_, γ_03_, γ_04_, γ_05_, γ_06_, and γ_07_ are the slope coefficients of *Year*_*i*_, *Flock*_*i*_, *Age*_*i*_, *Dage*_*i*_, *Sex*_*i*_, *Bs*_*i*_ , and *F*_*i*_, which explained the incremental change in the dependent variable for each unit of change in the independent variables.

To assess how inbreeding influences the important economic traits of IMCGs, it is helpful to design a proper breeding scheme. Referring to corresponding studies (14), inbreeding coefficients (*F*_*i*_) obtained by pedigree information were classified into four levels (*F*_*i*_ = 0; 0 < *F*_*i*_ ≤ 6.25; 6.25 < *F*_*i*_ ≤ 12.5; *F*_*i*_≥12.5). Variance analysis of inbreeding levels on each trait was performed by the aov function of R language.

## Results

### Analysis of Genetic Diversity and the Population Structure

**Figure 1:**
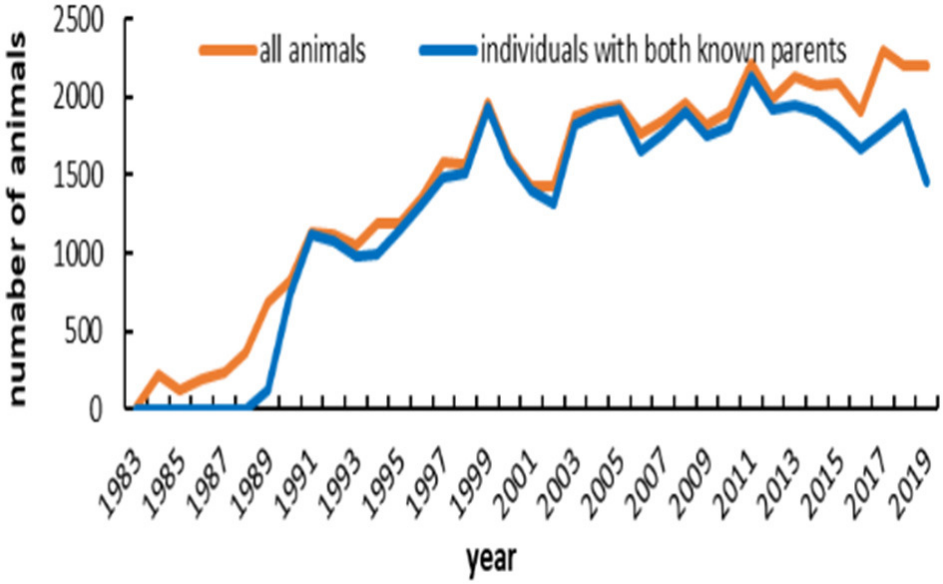
Number of animals and number of individuals with both known parents across years.

**Figure 2:**
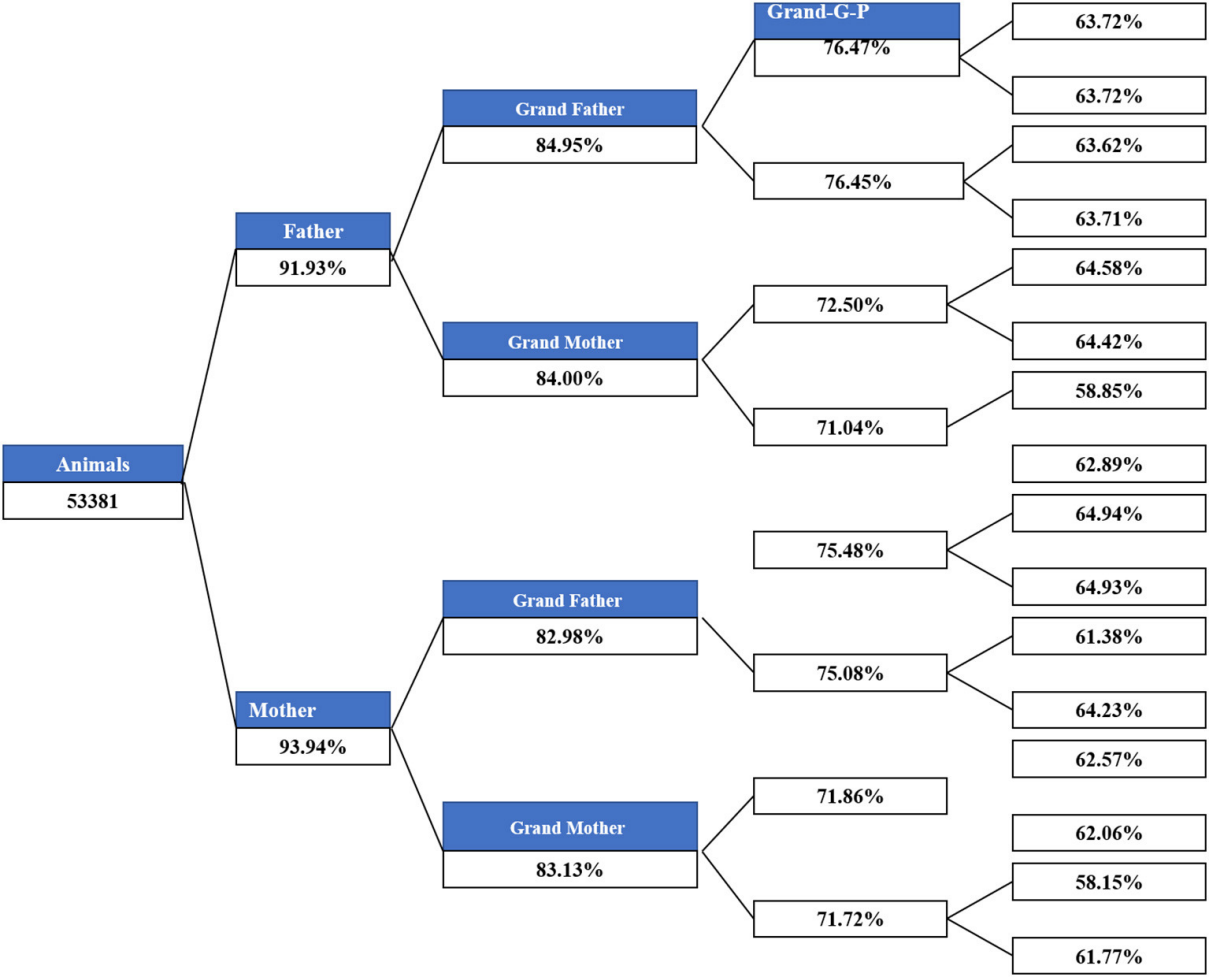
Pedigree completeness across generations.

The main demographic characteristics derived from genealogical data of IMCG flocks are summarized in Table 2. In this population, 0.8 and 20.5% are presented as sires and dams, respectively. Out of 53,381 animals, the number of founders (with one or more unknown parents) was 5,455, accounting for ~10.22%. Of the total animals, the ratio of individuals with no progeny was ~78.7%. The timeline trend of all animals and individuals with both known parents across years is presented in Figure 1. From 1983 to 1989, all animals in the pedigree were founders. The number of individuals with both unknown parents was relatively low from 1990 to 2012. After 1998, the number of individuals remained stable on this farm. Additionally, the pedigree completeness is shown in Figure 2. In total, of 53,381 animals, more than 90% had records with both parents. However, pedigree completion was poor when tracing back generations.

**Table 2:** Pedigree structure of Inner Mongolia White Cashmere goats.

**Item**	***N***
Individuals in total	53,381
Males	25,032
Females	28,349
Sires in total	433
Dams in total	10,923
Base population	5,455
Individuals with known sire	49,274
Individuals with known dam	51,095
Individuals with both unknown parents	114
Individuals with both known parents	47,957
Individuals with no progeny	42,027

**Figure 3:**
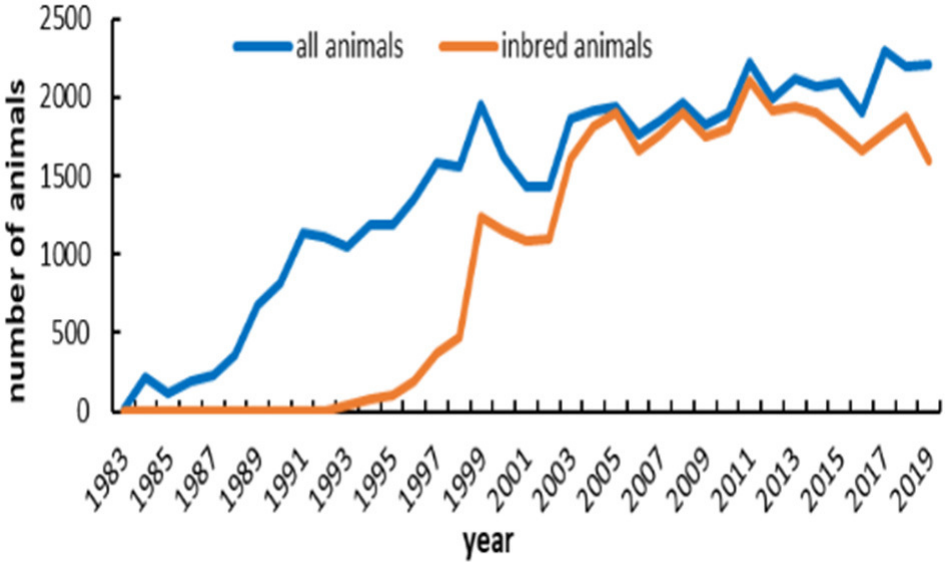
Registered and inbred individuals of IMCGs from 1983 to 2019.

**Figure 4:**
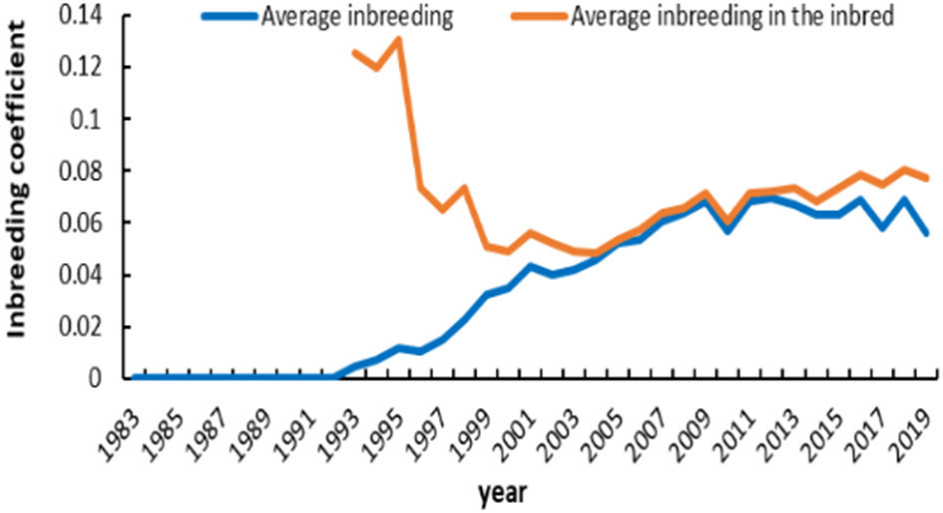
Average inbreeding coefficient in registered and inbred individuals in IMCGs across years.

The number of registered and inbred animals across birth years is shown in Figure 3. No inbred animal was found until 1993. The proportion of inbred animals in all registered individuals was more than 63.4%. An overall increasing trend did exist in the inbred animals for IMCGs from 1999 to 2005 and then remained stable. The number of inbred animals even reached 97.7% of the registered animals in 2005. The timeline trend of the average inbreeding coefficients is presented in Figure 4. Increasing trends of average inbreeding coefficients were observed in all registered animals. However, there was no regular trend for average inbreeding coefficients in the inbred animals. A decreasing trend for average inbreeding coefficients was observed from 1993 to 1998 in the inbred animals.

The probabilities of gene origin parameters in the studied breed are presented in Table 3. Of the 8,592 animals in the reference population, there were 4,298 males and 4,303 females. The number of founder animals in the reference population was 1980. The number of ancestors contributing to the reference population of IMCGs was 1977, which accounted for 99.8% of founders. The effective numbers of founders and ancestors in the studied population were 27.87 and 23.42, respectively. The effective numbers of founders and ancestors in the reference population were 24 and 19, respectively. The parameters of mean maximum generations, complete generations and equivalent generations were 11, 7, and 8.55, and the increases in inbreeding in the corresponding generations were 0.76, 1.66, and 1.30%, respectively. Fifty percent of genetic diversity was explained by six influential ancestors in the reference cohort. The founder genome equivalent of the reference population was 11.78. *F*-statistics were used to assess genetic differentiation in subdivided populations, and the value was 5.7 × 10^−5^.

**Table 3:** Parameters of the probability of gene origin for the reference population of IMCGs.

**Item**	**Value**
Number of animals in the reference population	8,592
Number of ancestors contributing to the reference population	1,977
Total number of founder animals in the reference population	1,980
Effective number of ancestors	23.42
Effective number of founders	27.87
Effective number of ancestors for the reference population	19
Effective number of founders for the reference population	24
Number of ancestors explaining 50%	6
Mean maximum generations	11
Increase in inbreeding by maximum generation	0.76%
Mean complete generations	7
Increase in inbreeding by complete generation	1.66%
Mean equivalent generations	8.55
Increase in inbreeding by equivalent generation	1.30%
Genetic conservation index	18.58
Wright *F*-statistics	5.7×10^−5^
Founder genome equivalent	11.78

The distribution of the inbreeding coefficient among IMCGs is presented in Table 4. A total of 36,602 animals were inbred. Approximately 55.7% of inbred animals had inbreeding lower than 6.25%. The inbreeding coefficient ranged from 0.98 to 35.93%. A total of 3.96% of inbred animals had an inbreeding coefficient > 25%. The average inbreeding value and mean average relatedness in the entire studied population were 4.50 and 8.48%, respectively. The average inbreeding in the inbred individuals was 6.57%. It is presumed that effective population size is considered the number of animals that breed in an ideal population and engender an equal amount of inbreeding in the population under study. The realized effective population size (Ner) was 15.16 ± 3.33.

**Table 4:** Inbreeding, average relatedness, and effective population size in IMCGs.

**Item**	**Value**
Coefficient of inbreeding (Fi) in the whole population (%)	4.50%
Proportion of animals with Fi = 0%	16,779 (31.43%)
Proportion of animals with Fi = 0 to ≤ 6.25%	20,402 (38.22%)
Proportion of animals with Fi = >6.25 to ≤ 12.50%	13,270 (24.86%)
Proportion of animals with Fi = >12.5%	2,930 (5.49%)
Average relatedness (AR%)	8.48%
Realized effective population size (Ner)	15.16 ± 3.33

This population has gone through 12 generations by pedigree tracing (Table 5). With the increase in generations, the inbreeding coefficient and average relatedness coefficient show an overall increasing trend. More than 90% of the individuals in the population are inbred in the fifth generation. The mean relatedness coefficient ranged from 9.49 to 10.74% after the fifth generation, which was relatively high. In the first two generations, there were no inbred animals, and the average relatedness was also very low, with values of 0.05 and 1.79%. The estimation of generation intervals (in years) for the four pathways of the IMCGs is presented in Table 6. The average generation length obtained in the present study was 4.11 ± 0.01 years. The ram-to-son pathway was lowest (3.97 years), and the ewe-to-daughter pathway was highest (4.24 years). However, no significant differences in generation intervals were observed among the four pathways in IMCGs.

**Table 5:** Mean value of inbreeding (*F*) and percentage of endogamic animals of IMCGs using the maximum number of generations traced.

**Generation**	**Animals (*N*)**	***F* (%)**	**% Inbred**	**Average *F* for inbred**	**Mean AR**
0	2,086	0.00%			0.05%
1	3,604	0.00%			1.79%
2	3,845	0.82%	6.50%	12.55%	4.62%
3	4,431	2.12%	30.17%	7.04%	6.56%
4	4,635	3.39%	67.92%	4.99%	8.24%
5	3,737	4.57%	90.71%	5.04%	9.49%
6	5,385	5.09%	94.97%	5.36%	9.85%
7	6,303	6.27%	96.02%	6.53%	10.83%
8	6,594	6.50%	93.13%	6.98%	10.74%
9	6,498	6.50%	87.12%	7.46%	10.42%
10	4,713	6.89%	88.71%	7.77%	10.54%
11	1,494	6.82%	85.88%	7.95%	10.40%
12	56	6.21%	80.36%	7.73%	9.73%

**Table 6:** Generation intervals (in years) for the four pathways of the IMCGs.

**Pathway**	***N***	**GI ± SE (years)**
Ram–Son	387	3.97 ± 0.07
Ram–Daughter	8,731	3.98 ± 0.01
Ewe–Son	387	4.03 ± 0.08
Ewe–Daughter	8,809	4.24 ± 0.02
Total	18,314	4.11 ± 0.01

### Effect of Inbreeding on Important Economic Traits of IMCGs

A linear regression model was established to assess the relationship between important economic traits and the inbreeding coefficients of IMCGs, and the results are shown in Table 7. The production year, herd, individual ages, age of dam, and sex had highly significant effects on all four traits (*P* < 0.01). The birth status had no significant effect on the FL and FD (*P* > 0.05), but the other three traits were significantly affected by the birth status (*P* < 0.01). Excluding the FL, the inbreeding coefficient had a significant effect on the other three traits. The results demonstrated that the LBW, CY, and FL increased by 3.88 kg, 208.7 g, and 1.151 cm, respectively, with every 1% increase in the inbreeding coefficient, and the FD decreased by 0.819 μm with every 1% increase in the inbreeding coefficient.

**Table 7:** Regression analysis of inbreeding on the economic traits of IMCGs.

**Effects**	**LBW**		**CY**		**FL**		**FD**	
	**Estimates**	***P*-value**	**Estimates**	***P*-value**	**Estimates**	***P*-value**	**Estimates**	***P*-value**
Intercept	34.92	< 0.01^**^	660.93	< 0.01^**^	5.899	< 0.01^**^	14.070	< 0.01^**^
Year	0.49	< 0.01^**^	13.22	< 0.01^**^	0.050	< 0.01^**^	0.067	< 0.01^**^
Herd	−0.12	< 0.01^**^	0.89	0.027^*^	−0.017	< 0.01^**^	−0.046	< 0.01^**^
Age	3.04	< 0.01^**^	4.96	< 0.01^**^	0.019	< 0.01^**^	0.136	< 0.01^**^
Dage	0.11	< 0.01^**^	−4.13	< 0.01^**^	−0.018	< 0.01^**^	0.012	0.013^*^
bt	−0.54	< 0.01^**^	10.64	< 0.01^**^	0.018	0.069^ns^	−0.009	0.591^ns^
Sex	−14.65	< 0.01^**^	−58.20	< 0.01^**^	−0.378	< 0.01^**^	−0.203	< 0.01^**^
F	3.88	< 0.01^**^	208.73	< 0.01^**^	0.151	0.178^ns^	−0.819	< 0.01^**^

*age, individual ages; dage, age of dams; bt, birth type; LBW, live body weight; CY, cashmere yield; FL, fiber length; FD, fiber diameter*.

** *highy signifficant;*

* *signifficant; ns, not signifficant*.

Variance analysis was performed to determine the threshold value of the inbreeding value for each trait. The results are shown in Table 8. Inbreeding levels had a significant effect on all of the traits (*P* < 0.01). Additionally, the multiple comparison analysis indicated that when the inbreeding coefficient was more than 6.25%, the LBW had an obvious decreasing trend. The threshold value of inbreeding depression in the CY is 12.5%. However, inbreeding depression has been observed in the FL and FD.

**Table 8:** Variance analysis of inbreeding levels on important economic traits of IMCGs.

**Traits**	**Class**	***N***	**Mean ± SD**	***P***
LBW	Fi = 0%	34,213	30.56, 8.56^c^	<0.01^**^
	Fi = 0 to ≤6.25%	19,020	37.65, 10.20^a^	
	Fi = >6.25 to ≤12.50%	7,965	37.35, 10.07^b^	
	Fi > 12.5%	2,019	37.06, 10.22^b^	
CY	Fi = 0%	34,856	550.0, 177.9^c^	<0.01^**^
	Fi = 0 to ≤6.25%	20,258	759.1, 211.6^a^	
	Fi = >6.25 to ≤12.50%	8,573	764.9, 208.3^a^	
	Fi > 12.5%	2,144	745.7, 204.8^b^	
FL	Fi = 0%	34,908	5.49, 1.00^b^	<0.01^**^
	Fi = 0 to ≤6.25%	20,434	6.18, 1.04^a^	
	Fi = >6.25 to ≤12.50%	8,649	6.21, 1.07^a^	
	Fi > 12.5%	2,157	6.19, 1.07^a^	
FD	Fi = 0%	2,051	14.76, 0.89^a^	<0.01^**^
	Fi = 0 to ≤6.25%	7,278	14.56, 1.00^b^	
	Fi = >6.25 to ≤12.50%	3,154	14.57, 0.99^b^	
	Fi > 12.5%	777	14.49, 0.97^b^	

** *highy signifficant*.

## Discussion

### Analysis of Genetic Diversity and Population Structure

In this population, 0.8 and 20.5% are presented as sires and dams, respectively. The fraction of males and females selected in IMCGs was relatively low. Individuals with no progeny accounted for 78.7% of all animals. All of these animals were sold as stud stocks to other flocks to improve performance or eliminated at the market for goat meat. Artificial insemination is used in this flock, so very few sires were selected. Out of 53,381 animals, the number of founders (with one or more unknown parents) was 5,455, accounting for ~10.22%. The results suggested a good depth in the pedigree in terms of completeness. Therefore, the accuracy of the estimated founders is reliable. The timeline trend of all animals with both known parents across the years indicated that the number of individuals with both unknown parents was low from 1990 to 2012; however, this value increased from 2013 to 2019. This may be related to incorrect pedigree information. After 1998, the population size remained stable. Our research team began to design a breeding plan for this flock in 1998, and thus, it had a reasonable population structure from then on.

This farm is representative of IMCGs. It has always undergone closed breeding and never introduced other breeds to cross. Therefore, the proportion of inbred animals among all registered individuals was more than 68.6%. A certain degree of inbreeding can improve production performance. Increasing trends of average inbreeding coefficients were observed in all registered animals. However, there was no regular trend for average inbreeding coefficients in the inbred animals. To maximize production performance, a reasonable mating plan is created every year. Most inbred animals showed inbreeding coefficients lower than 6.25%, and inbred animals with inbreeding coefficients > 25% accounted for ~4%. This may be related to our strict implementation of the proper mating scheme. A small fraction of high inbreeding individuals may be caused by natural mating or poor management. The average inbreeding value in the entire studied population was 4.50%, which is lower than that in the cashmere goat breed of the South Khorasan and Iranian Adani goat breeds reported by Joezy-Shekalgorabi et al. (34, 35). However, Illa et al. (14) reported that the average inbreeding coefficient in Nellore sheep was 3.32%, which was lower than that in the IMCG population. This difference may be explained by population structure, sample size, and management mode. The average relatedness in this population is 8.48%, which is higher than that in other ruminant breeds (36, 37). It is presumed that the effective population size is considered the number of animals that breed in an ideal population and engender an equal amount of inbreeding in the population under study. According to the FAO guidelines on preserving animal genetic resources, an effective population size of < 50 affects the fitness of the breed (38). The realized effective population size (Ner) noted in this population is 15.16 ± 3.33, which is far lower than that reported in Nellore sheep and Latxa dairy sheep (14, 39). This is caused by the differences among breeds, mating schemes, and breed plans. The *F*-statistic in this population is very low, which is far lower than that reported in other studies (37, 40). This indirectly reflects the genetic differentiation among the population. Thus, this result indicated that the genetic differentiation in the IMCG population was low.

Shortening the generation interval is one of the methods to increase genetic progress. It also may result in better economic returns. This outcome is the best choice for production enterprises. However, decreasing the ability of individuals to stay on the farm will intensify genetic variability losses, especially rams. The genetic contribution of those animals will be lower because of their short lifespan. The population in this study has gone through 12 generations by pedigree tracing. The average generation length obtained in the present study was 4.11 ± 0.01 years, which is longer than that estimated in Beetal goats. Tomar et al. (41) reported that the generation interval was 2.04 years for Beetal goats. Similar results were observed in other breeds of goats (34, 35, 42). The generation length is ~4 years for most goats. Compared to the sire–progeny pathways, a higher mean generation interval of the dam–progeny pathways was observed. This is probably attributable to the fact that breeding dams are usually kept for more years to produce offspring than sires. Similar results were obtained in Creole goats and Nellore sheep (14, 42). Animal conservation programs should balance the lowered generation intervals with decent annual genetic gains and breeding animals with sustained genetic variability on the farm.

### Effect of Inbreeding on Important Economic Traits of IMCGs

The linear regression analysis demonstrated that the LBW, CY, and FL increased by 3.88 kg, 208.7 g, and 1.151 cm with every 1% increase in the inbreeding coefficient, and the FD decreased by 0.819 μm with every 1% increase in the inbreeding coefficient. It was illustrated that inbreeding had no negative impact on the economic traits under the current mating scheme. However, the variance of analysis of inbreeding levels indicated that when the inbreeding coefficient of most individuals was higher than 6.25 and 12.5%, the LBW and CY will produce inbreeding depression, respectively. Hence, the breeding plan should be updated appropriately. However, it is surprising to find that the increase in inbreeding resulted in a decrease in the FD, which is beneficial to the improvement of fiber quality. The results of this population are similar to those reported by Dai et al. (43). This study illustrated that inbreeding depression for fleece traits did not exist when the inbreeding coefficient reached 12.5%. Most studies have shown that inbreeding has no significant effect on fleece traits (36). Sousa et al. (44) reported that inbreeding had a significant effect on the body weight of Anglo Nubiana breed goats. Vostra-Vydrova et al. (37) indicated that inbreeding coefficients showed a significant negative influence on milk performance in the White Shorthair goat breed. This may be due to the differences in the studied traits, population structure, and data size.

## Conclusion

Population structure across years in the Inner Mongolia Cashmere goat breed was documented in this study. A small number of unknown parents was found to be the reason for complete and detailed pedigree information. Although the proportion of inbred individuals in the entire population is high, the low average inbreeding coefficient and average relatedness obtained in the studied population indicated that the current mating scheme is relatively reasonable. Generally, animals with unknown parents are assumed to have no inbreeding. However, in reality, this will lead to underestimation of the inbreeding coefficient. An effective way to solve this problem is to ensure pedigree completeness. Alternatively, utilizing paternity testing methods helps to overcome the problem of pedigree incompleteness. Although inbreeding has not resulted in an obvious decrease in economic traits, it should be controlled properly when designing mating schemes.

## Data Availability Statement

The original contributions presented in the study are included in the article/supplementary material, further inquiries can be directed to the corresponding author/s.

## Author Contributions

TZ, XY, YY, and QL analyzed the data. RS, CD, and ZhiyW conceived of and coordinated the study. BM, RW, and JL helped in conceive of the study. ZhixW, YZhan, ZL, and YZhao help to collect data. ZhiyW and BZ wrote the manuscript. All authors read and approved the final manuscript.

## Conflict of Interest

TZ is only employed by the company Inner Mongolia Bigvet Co., Ltd. The remaining authors declare that the research was conducted in the absence of any commercial or financial relationships that could be construed as a potential conflict of interest.
